# Constrictive pericarditis requiring pericardiectomy: an unusual first presentation of extra-articular rheumatoid arthritis—a case report

**DOI:** 10.1093/ehjcr/ytae428

**Published:** 2024-08-19

**Authors:** Chinelo Egwu, Malo Scullion, Christopher Gingles, Sean Kerrigan

**Affiliations:** Cardiology Department, Forth Valley Royal Hospital, Larbert FK5 4WR, UK; Cardiology Department, Forth Valley Royal Hospital, Larbert FK5 4WR, UK; Cardiology Department, Forth Valley Royal Hospital, Larbert FK5 4WR, UK; Rheumatology Department, Forth Valley Royal Hospital, Larbert FK5 4WR, UK

**Keywords:** Case report, Constrictive pericarditis, Rheumatoid arthritis, Extra-articular rheumatoid, Pericardiectomy

## Abstract

**Background:**

We report an unusual case of rheumatoid arthritis presenting for the first time with pericardial constriction and bilateral pleural calcification, in the absence of prior articular disease.

**Case summary:**

A 46-year-old Caucasian male, who initially presented with shortness of breath, intermittent chest tightness and general malaise, underwent extensive diagnostic workup over a period of six months involving multiple hospital admissions. He was found to have pericardial constriction on echocardiogram and ultimately required surgical pericardiectomy due to decompensation. After multiple diagnostic tests and specialist opinion, the aetiology of pericardial disease was ultimately confirmed to be extra-articular rheumatoid disease without synovitis.

**Discussion:**

Significant pericardial constriction can occur as the initial presentation of rheumatoid disease and anti-CCP is a highly specific confirmatory test. Pericardial pathological specimen can be unhelpful in determining this aetiology, and constrictive physiology can occur due to chronic inflammation/fibrosis in the absence of significant calcification.

**Learning points**

- Rheumatoid arthritis should be considered as a cause of pericarditis/pericardial constriction even in the absence of articular symptoms.
- Anti-CCP is highly specific for rheumatoid arthritis.
- Pericardial pathology can be unhelpful in identifying underlying aetiology of pericardial disease.
- Constrictive physiology can occur in the absence of significant pericardial calcification.

## Introduction

Pericardial inflammation in the form of pericarditis, pericardial effusion (including complication by cardiac tamponade) and pericardial constriction, are well recognized extra-articular manifestations of rheumatoid arthritis.^1,2^ Between 30% and 50% of patients with rheumatoid arthritis have evidence of asymptomatic pericardial involvement in echocardiography or post-mortem studies, however symptomatic pericardial disease even amongst severe cases is much less prevalent (<10%).^1^ Typically, patients have an established rheumatoid arthritis diagnosis or have synovitis that precedes presentation with pericardial involvement.^1^

Constrictive pericarditis is the result of pericardial inflammation and thickening, which leads to loss of elasticity and thus external impedance that disrupts diastolic ventricular filling.^3,4^ Common causes in the developed world include cardiac surgery, chest radiotherapy, post-infectious, and inflammatory diseases.^2–5^ Treatment for symptomatic cases often involves initial conservative anti-inflammatory medical therapy, with surgical excision of the pericardium reserved for resistant cases or cases where there is established irreversible calcification of the pericardium.^2,5^

## Summary figure

**Figure:**
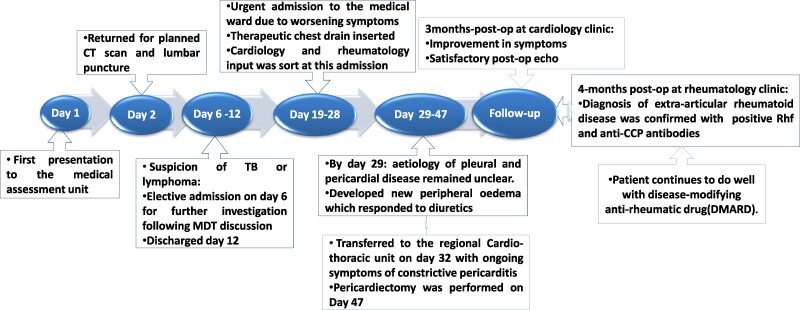


## Case presentation

A normally fit and well 46-year-old Caucasian male, with no relevant past medical history, presented to our medical assessment unit due to progressive shortness of breath over a six-week period. Respiratory rate was 28 breaths per minute, saturations 96% on air, blood pressure 130/90 mmHg, heart rate 75 beats per minute, and temperature 36.5°C. There were palpable lymph nodes in the neck. He had normal heart sounds, no murmurs, pericardial friction rub, or a raised jugular venous pressure. There was dullness to percussion bilaterally, predominantly on the left, and reduced air entry to the left lung. He had a palpable liver edge with otherwise unremarkable abdominal exam. There was no rash or joint swelling.

**Figure 1:**
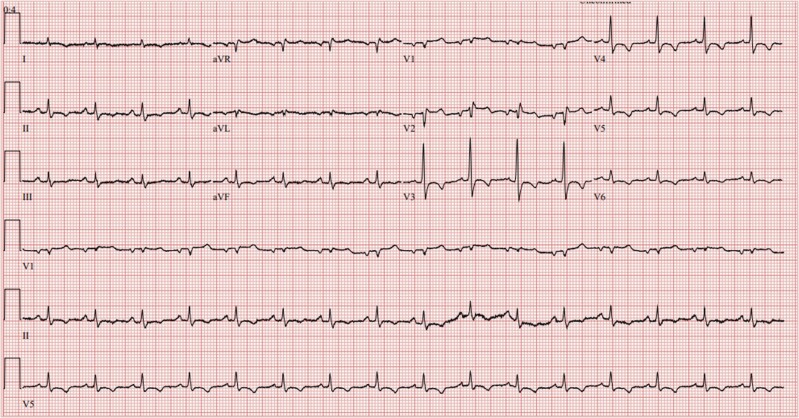
Electrocardiogram showing sinus rhythm with widespread T wave inversion.

Baseline blood tests showed a raised C-reactive protein 42 mg/L (ref. range <5 mg/mL) and erythrocyte sedimentation rate 38 mm/h (normal range for patients age and sex < 10 mm/h), normal troponin and otherwise unremarkable full blood count, and renal function tests. Twelve-lead electrocardiogram revealed sinus rhythm with widespread T wave inversion (*Figure 1*).

**Figure 2:**
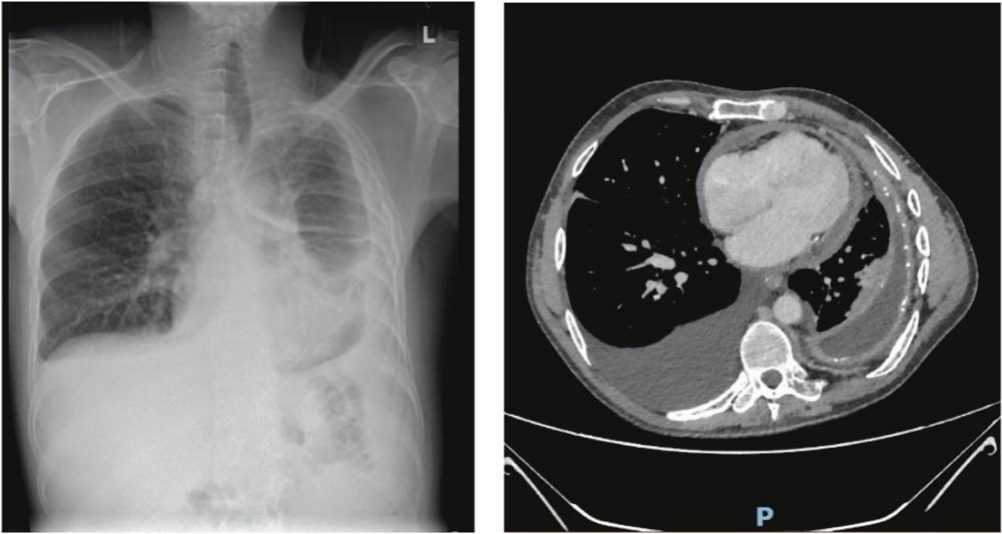
Index admission chest radiograph (*A*) demonstrating bilateral pleural effusions and left mid/lower zone opacification. (*B*) Axial slice of contrast computed tomography scan demonstrating rim enhancing pericardial effusion and bilateral pleural effusion with left-sided pleural calcification.

Admission chest X-ray demonstrated bilateral pleural effusions with extensive mid and lower zone opacification on the left (*Figure 2*). He was discharged with a plan for computed tomography chest/abdomen/pelvis (CT CAP).

Day 2: CT CAP revealed diffuse left-sided pleuro-pericardial thickening and enhancement with associated pleural calcification on the left (*Figure 2*). A large right-sided effusion was also seen.

**Figure 3:**
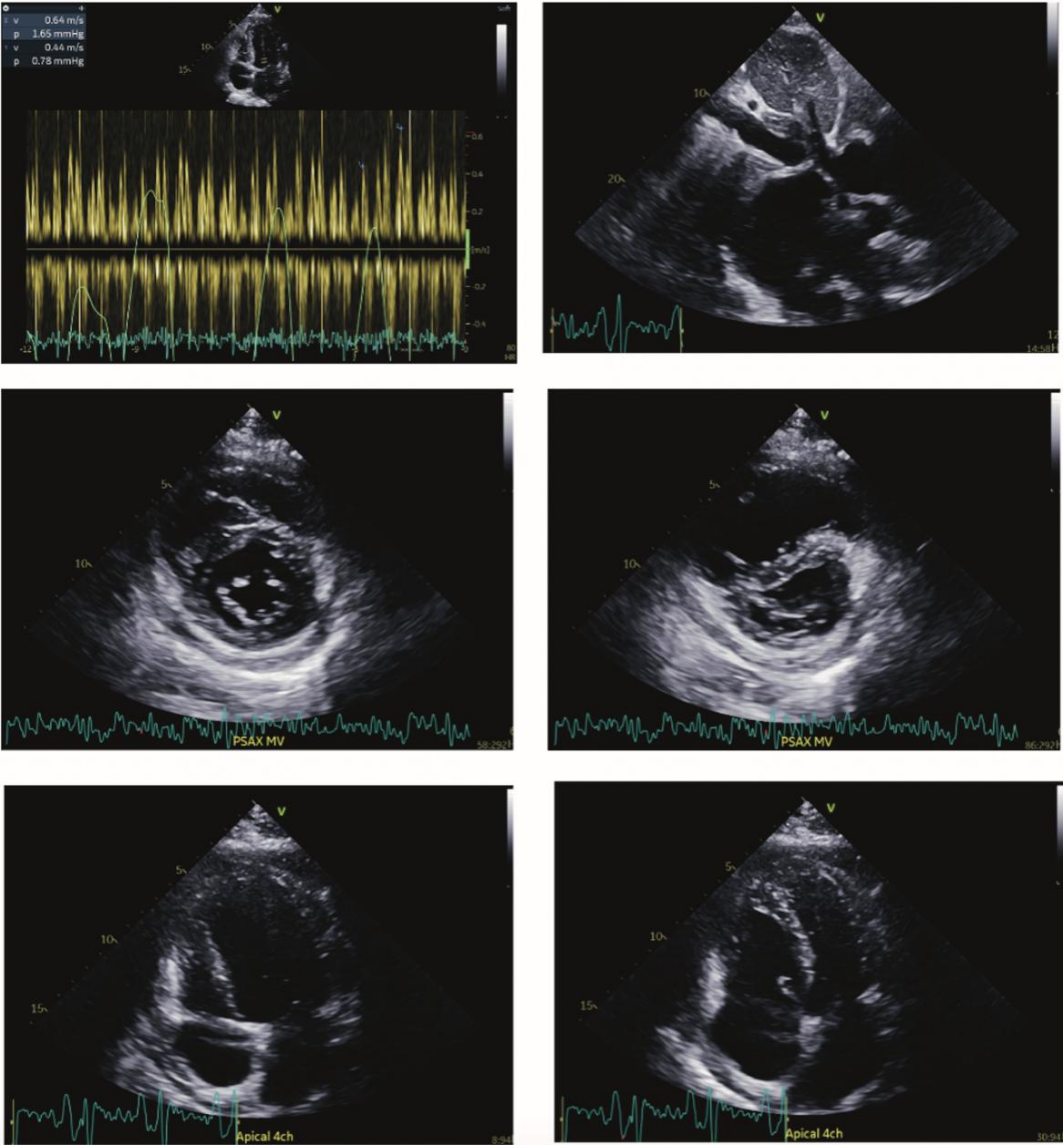
Index transthoracic echocardiogram demonstrating mitral valve inflow variation (*A*), inferior vena cava dilatation (*B*), and septal bounce (*C–F*).

Results of hepatitis, HIV screen, carcinoembryonic antigen, and prostate specific antigen was negative. An echocardiogram revealed features consistent with pericardial constriction/interventricular dependence including marked septal shift with respiration, annulus reversus mitral inflow variation, a dilated inferior vena cava, and a thickened bright pericardium with a trivial pericardial effusion (*Figure 3*). The atria were normal sized, and mitral valve inflow E/A ratio was normal. There were no significant valvular disease and insufficient tricuspid regurgitation to measure pulmonary artery systolic pressure.

His case was consulted with our cardiology team. He was clinically well at the time, we initiated colchicine and a non-steroidal anti-inflammatory drug for treatment of pericardial constriction and discharged with Cardiology/Rheumatology follow-up. Pleural and lymph node biopsy was also arranged.

The patient was readmitted to the hospital with worsening shortness of breath, new lower limb oedema, and general malaise. Repeat chest X-ray showed a worsening right-sided pleural effusion.

He underwent right-sided pleural drainage; continued anti-inflammatory agents and diuretic. Furosemide was titrated according to the clinical picture through the course of the hospital stay. Autoimmune assay results were now available. Serum angiotensin converting enzyme (ACE), P-ANCA, C-ANCA, anti-deoxyribonucleic acid antibody (anti-DNA), extractable nuclear antigen (ENA) antibodies, and complement were all negative. Anti-nuclear antibody (ANA) was mildly positive with 1/320 titre and a speckled pattern (normal range is a negative result at 1:80 screening dilution). Immunoglobulin A and G levels were elevated. The patient was reviewed by our rheumatology team who felt systemic lupus erythematosus was unlikely.

Despite conservative interventions, the patient’s condition deteriorated and he developed progressive pulmonary congestion and peripheral oedema. He was discussed at our regional multi-disciplinary meeting and referred to the cardiothoracic surgeons for urgent surgical pericardiectomy. Classical appearances of constrictive pericarditis were found intra-operatively with no calcification. There were no immediate complications, and the entire heart was free of constriction at the end of the procedure.

**Figure 4:**
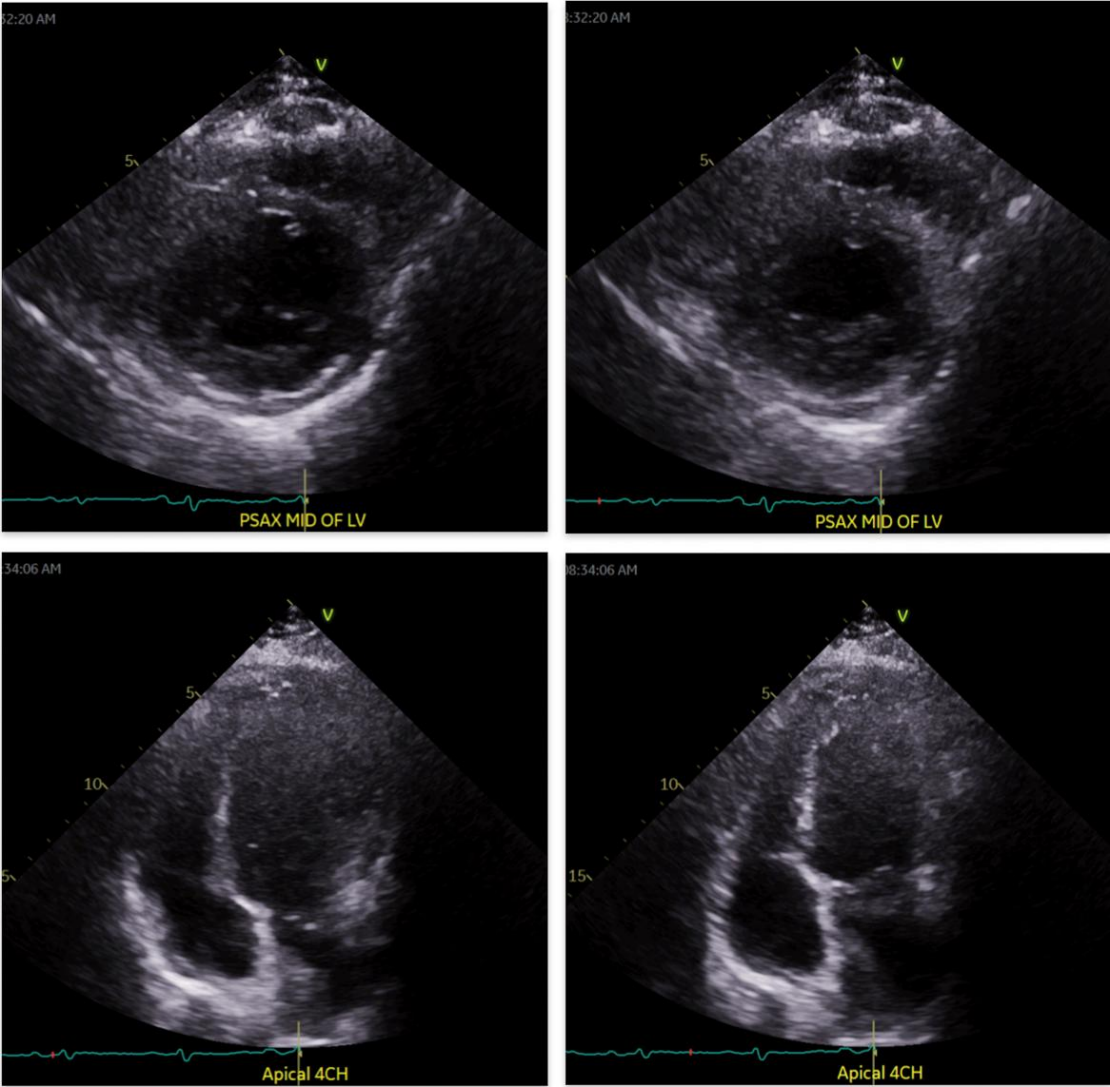
Post-operative echocardiogram showing diastolic (left) and systolic (right) phases with resolution of septal bounce.

At cardiology follow-up three months later, the patient was symptom free, and repeat echocardiography revealed normalization of left ventricular function with no further pericardial constriction (*Figure 4*). Histology results from the patient’s lung biopsy showed no malignant cells, and extended culture was negative for mycobacterium. Similarly, lymph node biopsy ruled out malignant cells. Histology from the surgically removed pericardium revealed chronic inflammation and fibrosis only. Periodic acid-Schiff-diastase stain was negative for Whipple’s disease.

**Figure 5:**
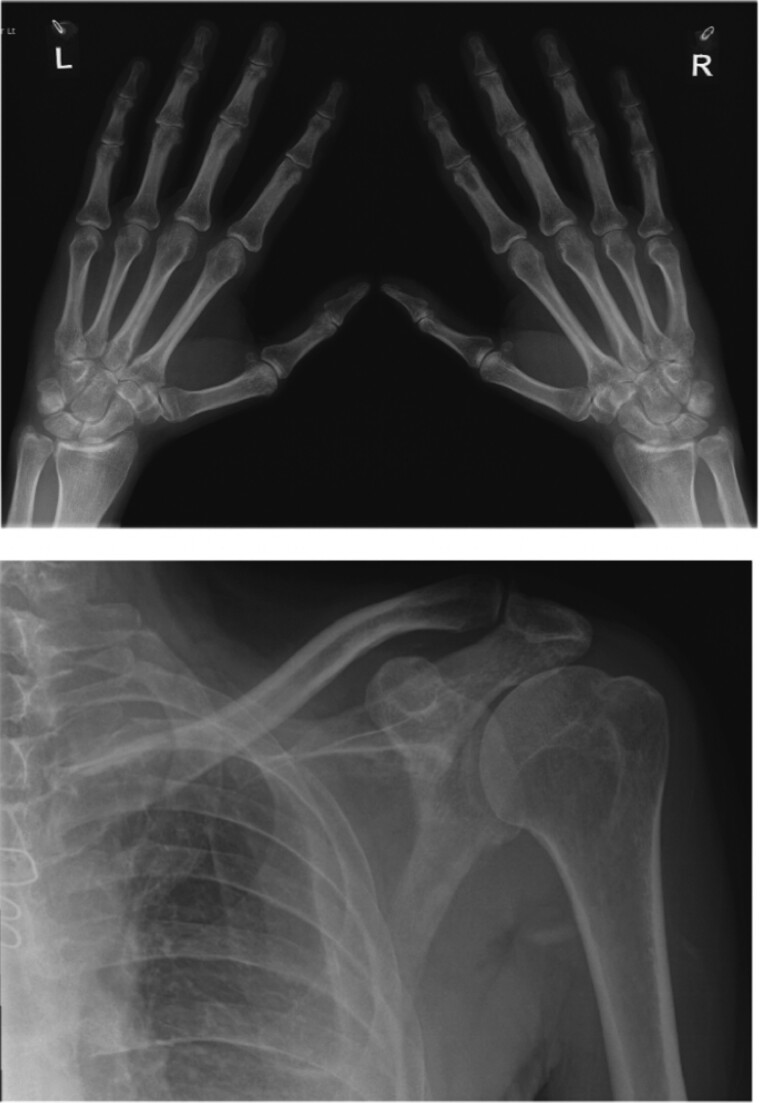
Radiographs of hands and left shoulder showing no evidence of erosive damage.

At rheumatology follow-up, a detailed history and re-assessment identified that 5 years prior to his presentation, he reported spontaneous shoulder pain and underwent physiotherapy with eventual resolution. This raised the possibility of previous episode of inflammatory joint disease. There were no significant articular symptoms or synovitis at the time of assessment. Further serological testing was negative for immunoglobulin G4 (IgG4) and syphilis. It did however reveal a strongly positive rheumatoid factor, 101 units/mL (ref. range: normal: 0–29 IU/mL, weak positive: 30–90 IU/mL) and anti-cyclic citrullinated peptide (anti-CCP) antibodies, >340 units/mL (ref. range 0–7 U/mL). This finally confirmed a diagnosis of rheumatoid arthritis, with pericardial inflammation as an extra-articular manifestation of the condition. X-rays of the hands and shoulder did not show evidence of erosive damage (*Figure 5*). The patient was commenced on methotrexate and a reducing course of prednisolone. Methotrexate was continued for a year thus far. The ongoing duration and dosage will be regularly reviewed and will depend upon clinical disease activity. Treatment holiday would have to be carefully considered given lack of joint symptoms on which to base assessment of active rheumatoid disease. Patient was given a prednisolone reducing regimen over 3–6 months.

## Discussion

Pericarditis as a predominant presenting feature of rheumatoid arthritis is rare, with only a small number of reports in the literature, and in most cases, synovitis was evident within a few months after diagnosis.^6^ To our knowledge, established pericardial constriction as the initial presentation of rheumatoid arthritis has only been reported in one previous instance in the setting of rheumatoid vasculitis, a severe manifestation of extra-articular rheumatoid.^7^ We report a further case of rheumatoid arthritis presenting with advanced pericardial constriction and bilateral pleural calcification, in the absence of any preceding known articular disease, which ultimately required pericardiectomy. Rheumatoid arthritis was not thought likely during initial workup given the lack of synovitis. The diagnosis of rheumatoid arthritis was eventually confidently made in this case due to a strongly positive anti-CCP, which is highly specific (95–99%) for rheumatoid arthritis even in the absence of typical synovitis.^8^ We note that the European Society of Cardiology 2015 Pericardial Diseases guidelines do not specifically recommend anti-CCP as part of the workup for pericardial disease thought autoimmune in origin, but they do recommend targeted search of underlying aetiology guided by specialist consultation.^2^

Pericardial inflammation due to rheumatological disease is managed with anti-inflammatory medication targeting the underlying rheumatological disease process. This typically involves initial glucocorticoid therapy and disease-modifying anti-rheumatic drugs (DMARD), as in this case.^9^ We recognize that early identification and targeted treatment of underlying aetiology of pericardial inflammation are important and could avoid the need for surgery. However, in this case, there was a short time period between first presentation, at an advanced stage, and decompensation requiring urgent pericardiectomy. We believe it unlikely in this case that initiation of DMARDS would have avoided surgery.

The main differential to exclude in the workup of suspected constrictive pericarditis is restrictive cardiomyopathy.^2,10^ The Mayo Clinic has identified three key independently associated echocardiographic variables in the identification of pericardial constriction^10^ The combination of ventricular septal shift and medial e′ > 9 cm/s, as in our case, is highly sensitive and specific (87% and 91%) for pericardial constriction.^10^ The European Society of Cardiology 2015 Pericardial Diseases guidelines recommend diagnosis of constrictive pericarditis in the presence of signs and symptoms of right heart failure and constrictive physiology demonstrated by one or more imaging methods including transthoracic echocardiography, CT, and cardiac MRI.^2^ We did not pursue invasive haemodynamic testing in this case given the combination of convincing echocardiographic constriction, extensive pericardial enhancement on CT, and lack of restrictive features.

Interestingly, a pericardial pathological specimen was unhelpful in determining the aetiology in this case. There was also no significant calcification on histological analysis of the pericardium. However, the marked chronic inflammation and fibrosis present explain the resultant constrictive physiology. While pericardial calcification is often associated with pericardial constriction, it is not always present.^11^

## Supplementary Material

ytae428_Supplementary_Data

## Data Availability

The data underlying this article are available in the online Supplementary material.

## Lead author biography

Dr Chinelo N. Egwu worked as clinical development fellow in cardiology at Forth Valley Royal Hospital, Larbert, and is currently a general practice registrar in the South East of Scotland Deanery. She is interested in the primary prevention of cardiovascular disease and heart failure management.

## Supplementary material

**Consent:** The authors confirm that written informed consent for patient information and images to be published was obtained in line with COPE guidance.

**Funding**: None to declare.
